# Supplementary material for: Development of a Time-Resolved Fluorescent Microsphere Test Strip for Rapid, On-Site, and Sensitive Detection of Picoxystrobin in Vegetables
Source: Foods. 2024 Jan 28;13(3):423. doi: 10.3390/foods13030423 (PMC10855143; doi:10.3390/foods13030423)
Supplement: Supplementary file 1 [file foods-13-00423-s001.zip › foods-2817640-supplementary material.pdf]

Development of a Time-Resolved Fluorescent Microsphere Test Strip for Rapid, On-Site, and Sensitive Detection of Picoxystrobin in Vegetables

Junjie Chen1, †, Lidan Chen,1, † Yongyi Zhang 1, Siyi Xiang 1, Ruizhou Zhang 1, Yudong Shen 1, Jiaming Liao 1, Huahui Xie 1, Jinyi Yang1, 2*

1 College of Food Science, South China Agricultural University, Guangdong Provincial Key Laboratory of Food Quality and Safety, Guangzhou 510642, China

2 Wens Institute, Wens Foodstuff Groups Co. LTD., Yunfu, P. R. China

† Both authors contributed equally to this work.

* Corresponding authors. E-mail addresses: yjy361@163.com，Tel: +86-20-8528-3925. Fax: +8620-8528-0270

Synthesis of PIC-H

LiOH 593 mg (14.1 mmol) and Bu_4NHSO_4 479 mg (1.41 mmol) were dissolved in 14 mL THF/H2O (3:1) solution.Then, at room temperature, PIC 518 mg(1.41 mmol) was added to the solution to react for 16 hours.After the reaction, the PH was adjusted to about 3.0 with $NaHSO_4$, followed by extraction with ethyl acetate.The organic layer was dried using anhydrous Na_2SO_4. Then, the solvent was removed under rotary evaporation.The crude mixture was purified by silica column elution (EA: PE = 1:5)to obtain intermediate product.

Next, 414 mg (1.5 mmol) of 6-bromohexanoic acid was dissolved in 2 mL DMF. The intermediate product (500 mg, 1 mmol), anhydrous K_2CO_3 (391 mg, 2 mmol), and triethylamine (295μL, 1.5 mmol) were dissolved in DMF.The solution of 6-bromohexanoic acid was added dropwise while stirring at 60°C overnight. After completion of the reaction, 50 mL distilled water was added, followed by extraction with ethyl acetate.After removing water with anhydrous Na_2SO_4, rotary evaporation of DMF yielded a yellowish oily substance.The oily substance was purified by silica

column elution (EA: PE = 1:1), followed by rotary evaporation of organic solvents, resulted in the final product, which is the PIC hapten(PIC-H).Hapten PIC-H was confirmed by electrospray ionization mass spectrometry (ESI-MS) analysis.

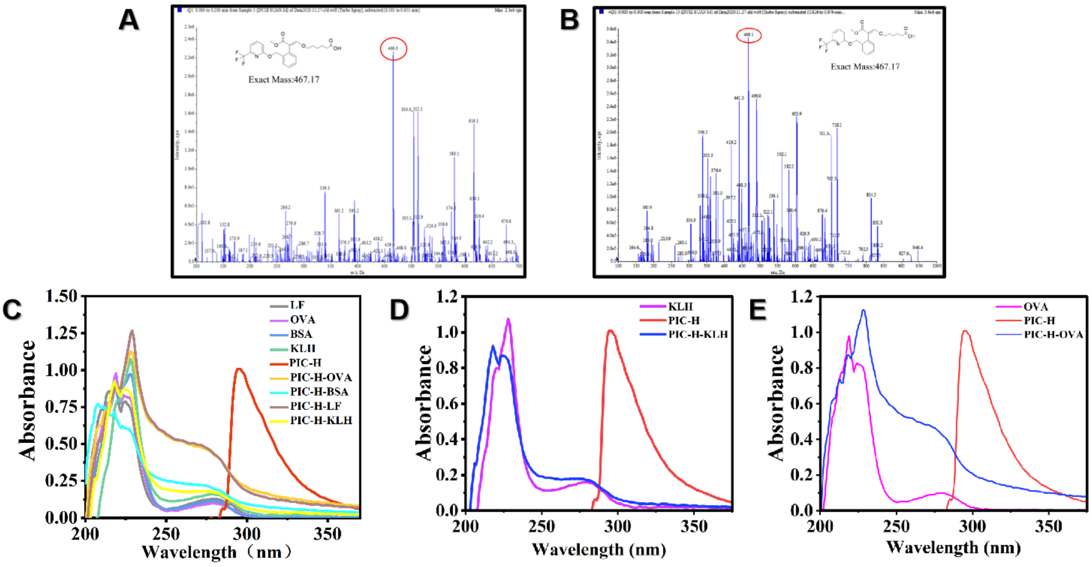

Figure S1. Characterization of hapten and artificial antigen. Hapten mass spectrogram (A)Negative spectrum and (B) Positive spectrum. (C)The UV–Vis spectroscopy of haptens, protein and conjugates. (D) Characterization of coating antigen. (E) Characterization of immunogen.

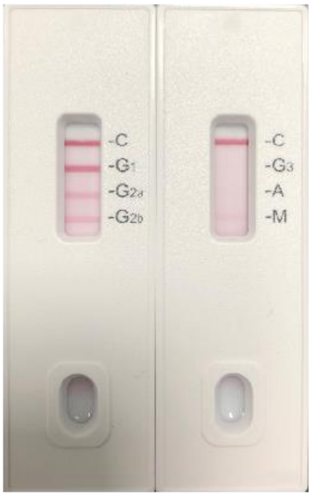

Figure S2 PIC16 ascites subtype determination chart.

Table S1 Cross reaction of TRFICA (n=3)

compound	structure	IC_{50} (ng/mL)	CR(%)
Picoxystrobin	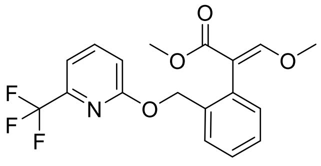	21.64	100
Fluazoxystrobin	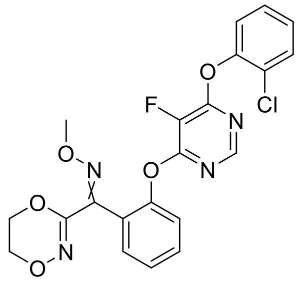	>2000	<1.1
Imazalil	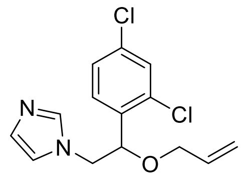	>2000	<1.1
Cyclopropiconazole	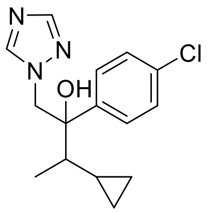	>2000	<1.1
Azoxystrobin	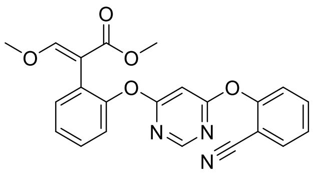	>2000	<1.1
pyraclostrobin	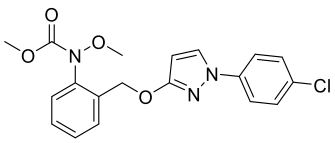	>2000	<1.1

Kresoxim-methyl	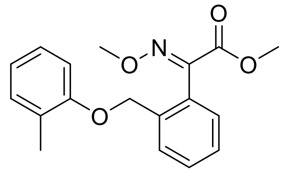	>2000	<1.1
DiMoxystrobin	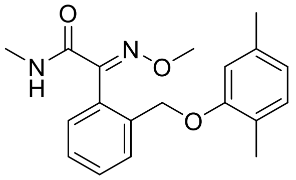	>2000	<1.1
